# Kinetic compartmentalization by unnatural reaction for itaconate production

**DOI:** 10.1038/s41467-022-33033-1

**Published:** 2022-09-12

**Authors:** Dae-yeol Ye, Myung Hyun Noh, Jo Hyun Moon, Alfonsina Milito, Minsun Kim, Jeong Wook Lee, Jae-Seong Yang, Gyoo Yeol Jung

**Affiliations:** 1 Department of Chemical Engineering, Pohang University of Science and Technology, 77 Cheongam-Ro, Nam-Gu, Pohang, Gyeongbuk 37673 Republic of Korea; 2 Centre for Research in Agricultural Genomics (CRAG), CSIC-IRTA-UAB-UB, Campus UAB, Bellaterra, Barcelona, 08193 Spain; 3 School of Interdisciplinary Bioscience and Bioengineering, Pohang University of Science and Technology, 77 Cheongam-Ro, Nam-Gu, Pohang, Gyeongbuk 37673 Republic of Korea

**Keywords:** Metabolic engineering, Synthetic biology, Applied microbiology

## Abstract

Physical compartmentalization of metabolism using membranous organelles in eukaryotes is helpful for chemical biosynthesis to ensure the availability of substrates from competitive metabolic reactions. Bacterial hosts lack such a membranous system, which is one of the major limitations for efficient metabolic engineering. Here, we employ kinetic compartmentalization with the introduction of an unnatural enzymatic reaction by an engineered enzyme as an alternative strategy to enable substrate availability from competitive reactions through kinetic isolation of metabolic pathways. As a proof of concept, we kinetically isolate the itaconate synthetic pathway from the tricarboxylic acid cycle in *Escherichia coli*, which is natively separated by mitochondrial membranes in *Aspergillus terreus*. Specifically, 2-methylcitrate dehydratase is engineered to alternatively catalyze citrate and kinetically secure *cis*-aconitate for efficient production using a high-throughput screening system. Itaconate production can be significantly improved with kinetic compartmentalization and its strategy has the potential to be widely applicable.

## Abstract (web summary)

Unlike eukaryotic system, bacterial hosts lack membranous system, which is one of the limitations for efficient metabolic engineering. Here, the authors report a kinetic compartmentalization strategy to increase substrate availability from competitive reactions for the efficient production of itaconate in *E. coli*.

## Introduction

Diverse intracellular biochemical reactions have evolved to efficiently supply energy and synthesize essential metabolites required for organism survival^1–4^. For example, highly orchestrated enzymatic reactions involving substrate channeling by multienzyme complex or consecutive enzyme reactions have evolved to rapidly consume intermediates, avoiding the formation of byproducts that undermine the efficiency of the desired pathway^5–9^. However, such an efficient metabolic reaction chain may limit metabolic engineering when the newly introduced metabolic pathway must use the metabolic intermediate as a substrate, as the accessibility of the intermediate is limited by kinetic competition with the native reaction chain^10^. For example, in itaconate biosynthesis, substrate availability can limit the biotechnological production of these valuable compounds^11^. Itaconate is a dicarboxylic acid used in the resin and plastic industry^12^ and can be synthesized from a decarboxylation reaction of *cis*-aconitate by *cis*-aconitate decarboxylase^11,13^. However, *cis*-aconitate is an intermediate metabolite that is transiently generated in the tricarboxylic acid (TCA) cycle via the enzyme aconitase, which converts citrate into isocitrate^8^. The strong affinity of aconitase enzyme for *cis-*aconitate causes its rapid conversion into isocitrate^8^, reducing the *cis*-aconitate availability for itaconate biosynthesis^11,13,14^.

One solution used by nature to avoid competing reactions is spatial compartmentalization^13,15,16^, which utilizes the physical separation of an intermediate to block kinetic competition among diverse chemical reactions^13,16^. For example, *Aspergillus terreus*, a native itaconate producer, facilitates spatial compartmentalization of *cis*-aconitate by pumping out mitochondrial *cis*-aconitate into the cytosol by exchanging cytosolic oxaloacetate via an antiporter, MttA (Supplementary Fig. 1)^17^. This physical separation allows the fungus to accumulate *cis*-aconitate in the cytosol and efficiently produce itaconate by avoiding competition with the TCA cycle. Further, itaconate production can be efficiently regulated via MttA activity^18^. However, because of the insufficiently implemented genetic engineering tools and complicated culture conditions of *A. terreus*, research focused on itaconate production has been performed in well-known workhorse cells such as *Escherichia coli*^11,14^. Mimicking the spatial compartmentalization in *E. coli* would be an efficient approach for itaconate production; however, prokaryotic hosts lack a membranous system, leading to challenges in building a compartmentalized system^11,14,15^.

In this work, we adapt a kinetic compartmentalization approach to overcome the inapplicability of spatial compartmentalization in bacteria. We introduce an unnatural enzymatic reaction to kinetically separate competitive reactions by releasing the intermediate from the native reaction chain without creating a physical barrier. Particularly, we kinetically compartmentalize the itaconate production reaction from the TCA cycle by introducing a non-natural enzyme that can separate consecutive biochemical reactions catalyzed by aconitase. We hypothesize that introducing a non-natural biochemical reaction into *E. coli* cells, which can effectively synthesize *cis*-aconitate from citrate, would increase the intracellular *cis*-aconitate level, thus triggering its conversion into itaconate through catalysis by *cis*-aconitate decarboxylase.

**Fig. 1:**
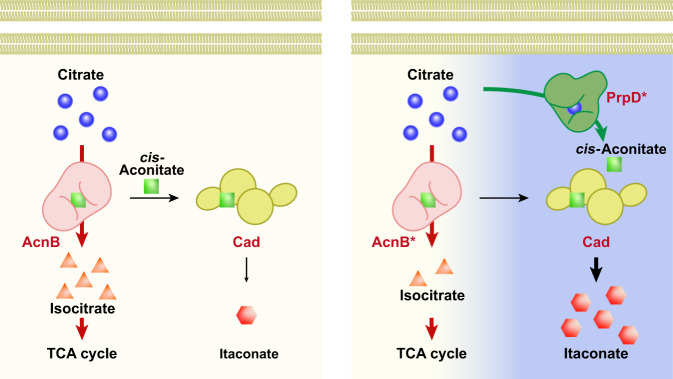
Schematic diagram of overall strategies for kinetic compartmentalization using the non-natural enzyme reaction. 2-Methylcitrate dehydratase (PrpD) was evolutionarily engineered to alter the substrate preference and catalytic efficiency towards citrate. The desired PrpD mutant has higher activity towards citrate to secure *cis*-aconitate, resulting in increased itaconate production. The non-natural enzyme exhibits a kinetically compartmentalized effect without actual spatial separation. Citrate (blue); isocitrate (orange); *cis*-aconitate (green); itaconate (red).

In particular, endogenous 2-methylcitrate dehydratase, PrpD^19^ is successfully engineered that originally converts 2-methylcitrate into 2-methyl-*cis*-aconitate, to catalyze the conversion of citrate into *cis*-aconitate. PrpD is evolutionarily engineered to switch its specificity from 2-methylcitrate to citrate by computational simulation design and high-throughput screening^20–22^. Based on the non-natural enzyme, an unnatural itaconate production pathway is constructed that is kinetically isolated from the TCA cycle (Fig. 1). Using our approach, we obtain a significantly increased itaconate yield of up to 10.6-fold compared to the parental strain at 46.0% of the theoretical maximum^14^. This implementation of kinetic compartmentalization in bacteria allows overcoming the limitation of being a spatially inseparable host by introducing an unnatural biochemical reaction able to efficiently separate competitive reactions. This method shows potential for kinetic compartmentalization in prokaryotes, considering the substantial number of promiscuous enzymes that can be engineered using this approach.

## Results

### Selection of target enzyme for compartmentalization

To separate *cis*-aconitate production from the TCA cycle, we first reviewed the detailed reaction mechanisms of *cis*-aconitate synthesis in *E. coli*. The precursor of itaconate, *cis*-aconitate, can be synthesized by aconitase encoded by *acnA* and *acnB* in *E. coli*. Dehydration of citrate is sequentially coupled to rehydration of *cis*-aconitate by the same enzymes, which appear to catalyze the isomerization of citrate to isocitrate. Thus, *cis*-aconitate is temporarily synthesized as a reaction intermediate (Supplementary Fig. 2a). Furthermore, the catalytic efficiency (*k*_cat_/*K*_m_) of aconitase is much higher for *cis*-aconitate than for citrate^8^. Consequently, *cis*-aconitate accumulates at very low levels in native *E. coli*^11,14^, resulting in low itaconate production. Aconitase may only conduct a dehydration reaction, however, the dehydration and rehydration reactions are coupled with two main catalytic residues, H444 and S244^23^. H444 functions as a proton donor, whereas S244 acts as a proton acceptor in the first dehydration reaction^23^. In the rehydration reaction, H444 acts as a proton acceptor, and S244 functions as a proton donor, switching the roles of these residues^23^. After these two reactions, the catalytic residues return to the initial state to conduct another round of reactions. Thus, it is difficult to isolate the dehydration reaction.

We screened enzymes with dehydration activity; their known substrates are similar to those of citrate structures. Among all enzyme candidates, the endogenous enzyme of *E. coli*, PrpD, converts 2-methylcitrate into 2-methyl-*cis*-aconitate in a one-step dehydration reaction without rehydration to 2-methylisocitrate (Supplementary Fig. 2b)^19^. This enzyme has a much lower affinity and catalytic efficiency for citrate and *cis*-aconitate compared to aconitases^19^. Engineering PrpD to alternatively catalyze citrate into *cis*-aconitate may be a useful strategy for enhancing the pool of *cis*-aconitate for itaconate production.

### Development of itaconate-specific screening system

**Fig. 2:**
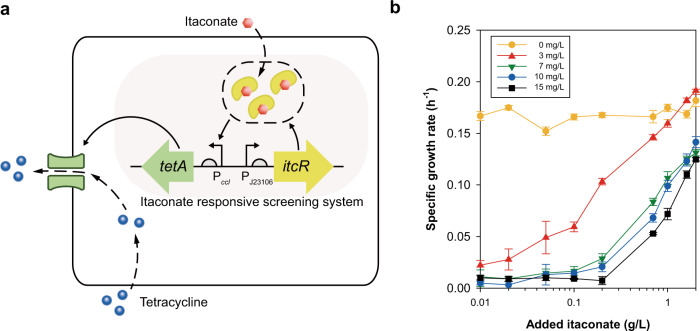
Design and validation of itaconate-responsive screening system. **a** Schematic diagram of itaconate-responsive screening system based on the tetracycline resistance gene. **b** Specific growth rates of WS strains were plotted in accordance with itaconate concentration under varied tetracycline concentrations. Data were presented as mean values and error bars indicate the standard deviations from three biological replicates. Source data are provided as a Source Data file.

Recently, an itaconate-responsive LysR-type transcription factor, ItcR, from *Yersinia pseudotuberculosis* was shown to regulate the expression of the itaconate degradation pathway based on the presence and amount of itaconate^24^. We exploited ItcR and its cognate promoter, P_*ccl*_, to construct an itaconate-responsive screening system. The screening system was designed to regulate the expression of the antibiotic resistance gene^25,26^ according to itaconate concentration. Specifically, ItcR was constitutively expressed using a synthetic promoter (P_BBa_J23106_), and the tetracycline resistance gene (*tetA*) was controlled under P_*ccl*_. Collectively, the system was intended to provide a growth advantage under tetracycline pressure in environments with high itaconate concentrations or in high-producing strains (Fig. 2a).

To validate the screening system, the itaconate-responsive screening system was transformed into an acid-tolerant *E. coli* W strain^14^ to produce the WS strain (Supplementary Data 1). We demonstrated the effectiveness of the screening system and a further improvement in itaconate production using a previously developed itaconate-producing *E. coli* strain with acetate as the sole carbon source^14^. The tetracycline concentration as the selection pressure was varied up to 15 mg/L and growth retardation were confirmed in accordance with the increased tetracycline concentration, indicating tight regulation of ItcR/P_*ccl*_ (Fig. 2b). In addition, the gradual increase in the specific growth rate was validated by extracellular addition of itaconate under selection pressure. Specifically, the specific growth rate increased as the concentration of itaconate increased to 2 g/L. Additionally, the growth rate according to itaconate showed different tendencies depending on the tetracycline selection pressure (Fig. 2b). Collectively, these results indicate that the itaconate-responsive screening system was successfully constructed and is widely applicable for itaconate production.

### Screening PrpD mutant with altered substrate specificity

To select enzyme variants that can produce *cis*-aconitate, PrpD protein engineering was conducted using the itaconate-responsive screening system. Target residues for mutagenesis were selected based on structural analysis of tartrate-bound MmgE (PDB code: 5MUX [https://www.rcsb.org/structure/5MUX]), the homolog of PrpD derived from *Bacillus subtilis*^27^. As the catalytic residues are all conserved in PrpD (Supplementary Fig. 3) and the tartrate has a similar structure with 2-methylcitrate, we hypothesized that the tartrate-bound structure could give useful information about substrate-residue interactions. Then we superimposed 2-methylcitrate on the l-tartrate and selected residues near the methyl group of 2-methylcitrate. As our objective is to find mutants to recognize citrate instead of 2-methylcitrate, we were looking for more hydrophobic and small residues. The methyl group of 2-methylcitrate in our superimposed model heading toward W110 and G111. These residues make a hydrophobic pocket with I331 that seems to allow to bind methyl group (Supplementary Fig. 4). We also confirmed that the methyl group of 2-methylcitrate toward W110 and G111 with our docking simulation (Supplementary Fig. 5). So we decided to mutate W110, G111, and I331 residues to switch the catalytic activity from 2-methylcitrate to citrate, which has no methyl group on the second carbon. A mutant library of PrpD (W110, G111, and I331 residues) was constructed for expression in moderate strength (BBa_P_J23108_) based on structural analysis (see Methods) with theoretical 20^3^ variants numbers in size and transformed.

To increase the intracellular level of citrate and ensure the activity of PrpD mutants, the catalytic efficiency of the competing enzyme, AcnB, was decreased through site-directed mutagenesis (AcnB^W482R^, Table 1)^28^. Mutagenesis of AcnB efficiently lowered the catalytic efficiency to citrate by 3.75-fold (Table 1). Notably, by knocking out the *iclR*-encoding transcriptional repressor for the glyoxylate shunt pathway, the pathway was activated to increase anaplerosis, ensuring the level of oxaloacetate, the different substrate for citrate, which is able to result in enhancing itaconate production^14,29^. The resulting WAIC strain (*E. coli* W (*acnB*_W482R) *ΔiclR* harboring *cad* expressing-plasmid (pCAD)) showed a lower cell biomass (2.16 g DCW/L) and itaconate production (0.26 g/L) by 1.17- and 1.22-fold, respectively, compared to the WCI strain (with wild-type *acnB*) (Supplementary Fig. 6)^14^. Nevertheless, the accumulation of citrate was significantly enhanced by up to 0.51 g/L, as expected. Overall, an environment was created in which the PrpD mutant with altered substrate specificity produced increased itaconate and showed a sufficient growth advantage in the selection condition. The screening was conducted by increasing the selection pressure from 7–15 mg/L of tetracycline over four rounds.

**Table 1:** Kinetic parameters of AcnB and AcnB^W482R^

Enzyme	Substrate	k_cat_ (s^−1^)	K_m_ (mM)	k_cat_/K_m_ (mM^−1^ s^−1^)
AcnB	Citrate	4,488.62 ± 696.56	3.32 ± 1.03	1,402.61 ± 249.94
AcnB^W482R^	Citrate	2,232.31 ± 758.05	6.34 ± 3.12	374.31 ± 67.74

Source data are provided as a Source Data file.

### Characterization of enriched PrpD mutants

After enrichment, ten isolated mutants were analyzed, and five types of mutants were characterized (Supplementary Table 1). We initially validated itaconate production for each mutant (WAICP^NNN^ strains, Supplementary Data 1) at the test tube scale (Supplementary Fig. 7). The WAICP^VTL^ strain (PrpD^VTL^ with W110V, G111T, and I331L) showed a 1.50-fold increase in itaconate production, whereas most mutants showed a decreased level of itaconate production compared to the WAICP strain with wild-type PrpD.

**Fig. 3:**
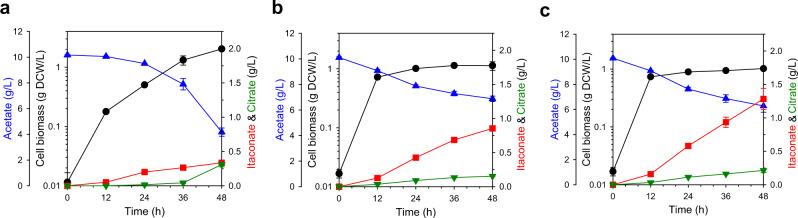
Fermentation profiles of itaconate production. **a** WAICP, **b** WAICP^VTL^, and **c** WAICP^VL^ strains. The left y-axis and y-offset represent the cell biomass (g DCW/L) and acetate (g/L), respectively. The right y-axis indicates the production of itaconate and citrate (g/L). The x-axis denotes time (h). Symbols: circles, cell biomass; up-triangles, acetate; squares, itaconate; down-triangles, citrate. Data were presented as mean values and error bars indicate the standard deviations from three biological replicates. Source data are provided as a Source Data file.

An additional culture was conducted at the flask-scale to validate the WAICP^VTL^ strain (Fig. 3a, b). Interestingly, the cell biomass was remarkably reduced in the WAICP^VTL^ strain (Fig. 3b) compared to that of the WAICP strain (Fig. 3a) by 1.80-fold (1.01 g DCW/L), and citrate accumulation was reduced by 2.00-fold (0.15 g/L), similar to that of the WCI strain (Fig. 3b and Supplementary Fig. 6a). These results indicate that PrpD^VTL^ exhibits increased catalytic efficiency toward the citrate to produce *cis*-aconitate, which induced kinetic separation and redirected more citrate for use in itaconate production. Indeed, the WAICP^VTL^ strain showed a 2.56-fold increase in itaconate production to 0.86 g/L compared to the WAICP strain. Moreover, the itaconate yield was significantly enhanced in the WAICP^VTL^ strain by up to 4.76-fold (0.27 g/g), indicating that a kinetic compartmentalization strategy is a powerful approach for enhancing the *cis*-aconitate availability and itaconate production.

To validate the effect of each residue on PrpD^VTL^, mutants at single residues and a combination of double mutants were generated as the WAICP^V^, WAICP^T^, WAICP^L^, and WAICP^TL^ strains (Supplementary Data 1); these mutants showed no significant difference in itaconate production compared to the WAICP strain (Supplementary Fig. 8). In contrast, the WAICP^VT^ and WAICP^VL^ strains showed 3.55- (1.19 g/L) and 3.84-fold (1.28 g/L) increases in the itaconate titer at 48 h (Supplementary Fig. 8 and Fig. 3c), respectively, which are even higher than that of WAICP^VTL^ (Fig. 3b). These results agree with those of structural analysis, as W110 is the closest residue among the target residues to the methyl group of the second carbon on 2-methylcitrate (Supplementary Table 2), combined with the rationale for the initial multi-residue library design.

### Kinetic and structural analysis of PrpD mutants

The enzyme kinetics of wild-type PrpD, PrpD^VTL^, PrpD^VT^, and PrpD^VL^ were characterized to evaluate the enhancement of itaconate production (Table 2). First, significant decreases of affinity to the natural substrate of PrpD, 2-methylcitrate, were observed in mutants as we desired. For example, PrpD^VTL^ showed the lowest affinity (1.96 mM of *K*_m_) toward 2-methylcitrate, which contributed to lowering the catalytic efficiency (0.013 mM^−1^ s ^−1^) by 5.77 times compared to the wild-type PrpD (0.075 mM^−1^ s^−1^). On the other hand, wild-type PrpD shows promiscuity for catalyzing citrate^19^ as demonstrated previously. However, this enzyme showed 12.21-fold lower catalytic efficiency compared to AcnB (Tables 1, 2), supporting the slight decrease in citrate and increase in itaconate of the WAICP strain (Fig. 3a) compared to the WAIC strain (Supplementary Fig. 6b). In contrast, the higher affinity for citrate (15.81 mM of *K*_m_) was confirmed in PrpD^VL^ compared to wild-type PrpD (66.39 mM), as expected. However, the mutant displayed a 1.35-fold lower turnover rate (*k*_cat_) for citrate (7,804.72 s^−1^), improving the catalytic efficiency by 3.11-fold (Table 2).

**Table 2:** Kinetic parameters of PrpD, PrpD^VTL^, PrpD^VT^, and PrpD^VL^

Enzyme	Substrate	k_cat_ (s^−1^)	K_m_ (mM)	k_cat_/K_m_ (mM^−1^ s^−1^)
PrpD	2-methylcitrate	0.045 ± 0.025	0.96 ± 0.85	0.075 ± 0.054
	Citrate	10,561.95 ± 3,427.82	66.39 ± 22.03	159.37 ± 2.36
	*cis*-Aconitate	119.03 ± 13.42	0.70 ± 0.10	170.61 ± 5.65
PrpD^VTL^	2-methylcitrate	0.024 ± 0.008	1.96 ± 0.21	0.013 ± 0.005
	Citrate	9,586.41 ± 1,193.47	35.32 ± 5.17	272.17 ± 11.72
	*cis*-Aconitate	81.83 ± 14.14	0.72 ± 0.10	113.60 ± 14.61
PrpD^VT^	2-methylcitrate	0.023 ± 0.008	1.93 ± 0.21	0.012 ± 0.006
	Citrate	6,093.22 ± 1,122.92	14.82 ± 2.38	410.08 ± 22.85
	*cis*-Aconitate	172.08 ± 19.98	1.72 ± 0.25	100.52 ± 3.18
PrpD^VL^	2-methylcitrate	0.035 ± 0.009	1.72 ± 0.54	0.021 ± 0.002
	Citrate	7,804.72 ± 693.10	15.81 ± 1.89	494.97 ± 15.22
	*cis*-Aconitate	96.34 ± 4.55	1.52 ± 0.08	63.63 ± 3.33

Source data are provided as a Source Data file.

A noticeable decrease in the affinity for *cis*-aconitate was also observed in PrpD^VL^ (1.52 mM of *K*_m_) compared to wild-type PrpD (0.70 mM, Table 2). The change in the binding pocket following mutagenesis may have altered the affinity for *cis*-aconitate and citrate^26,30^. In addition, the turnover rate of PrpD^VL^ was reduced (96.34 s^−1^), leading to a 2.68-fold decrease in catalytic efficiency. Collectively, these results indicate that PrpD^VL^ efficiently extracted citrate from the TCA flux and induced kinetic compartmentalization by converting it into *cis*-aconitate to facilitate itaconate production (Fig. 1).

Computational simulations of 2-methylcitrate into PrpD enzyme showed that in the wild-type, single mutated (PrpD^V^, PrpD^T^, and PrpD^L^), and double mutant (PrpD^TL^) enzymes, the methyl group faced the residues W110 and G111 (wildtype or mutated), while in the double mutants PrpD^VT^ and PrpD^VL^, and in the triple mutant PrpD^VTL^, the conformation shifted the methyl group in the opposite direction (Supplementary Fig. 5). Moreover, docking simulations revealed a slight decrease in hydrogen bonds between 2-methylcitrate and PrpD residues in the double mutants PrpD^VT^ and PrpD^VL^ and triple mutant PrpD^VTL^ compared to simulation into the active pocket of wild-type (Table 3). In contrast, docking predictions of citrate interactions showed an increased number of hydrogen bonds between this ligand and PrpD residues in all mutants compared to the bonds in the wild-type enzyme (Supplementary Fig. 9 and Table 3). Interestingly, mutants showing a higher substrate specificity for citrate (PrpD^VT^, PrpD^VL^, and PrpD^VTL^) exhibited a greater increase in hydrogen bonds between citrate and PrpD residues, with W110V potentially acting as the most important mutation determining the substrate shift, which is supported by the fact that mutating this single amino acid increased the number of hydrogen bonds of citrate compared to 2-methylcitrate, in the previously mentioned double and triple mutants (Table 3). Finally, docking on the double mutant PrpD^TL^ gave the same amount of hydrogen bonds with 2-methylcitrate and citrate, in accordance with the data showing the lowest enzymatic activity of this mutant towards citrate.

**Table 3:** Docking simulations summary

Receptor	Ligand	Binding energy (kcal mol^−1^)	H bonds
PrpD	2-Methylcitrate	−5.0	4
	Citrate	−5.0	4
PrpD^V^	2-Methylcitrate	−4.9	4
	Citrate	−4.8	6
PrpD^T^	2-Methylcitrate	−5.2	4
	Citrate	−5.1	5
PrpD^L^	2-Methylcitrate	−5.1	4
	Citrate	−5.0	5
PrpD^VT^	2-Methylcitrate	−4.9	3
	Citrate	−4.8	6
PrpD^VL^	2-Methylcitrate	−4.9	3
	Citrate	−4.8	6
PrpD^TL^	2-Methylcitrate	−5.1	5
	Citrate	−5.0	5
PrpD^VTL^	2-Methylcitrate	−4.9	3
	Citrate	−4.8	6

### Further flux optimization for increasing production

**Fig. 4:**
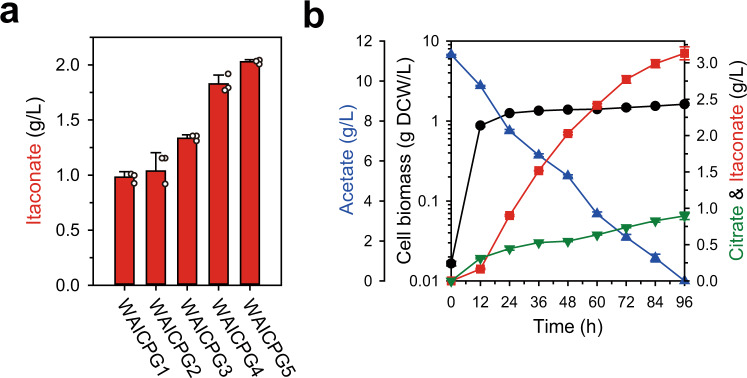
Further optimization of itaconate production. **a** Comparison of the itaconate titer of strains with varied *aceA* expression for 48 h. White dots indicate actual data. **b** Fermentation profile of WAICPG5 strain. The left y-axis and y-offset represent the cell biomass (g DCW/L) and acetate (g/L), respectively. The right y-axis indicates the production of citrate and itaconate (g/L). The x-axis denotes time (h). Data were presented as mean values and error bars indicate the standard deviations from three biological replicates. Source data are provided as a Source Data file.

The WAICP^VL^ strain was further optimized. First, PrpD^VL^ expression was optimized by employing constitutive promoters (Supplementary Fig. 10)^31^. Itaconate production was significantly affected by PrpDVL expression, as expected, indicating that efficient kinetic compartmentalization can allow metabolic flux to be regulated by changing the activity of PrpD^VL^ like spatial compartmentalization in *A. terreus*, where the precise flux distribution is regulated by MttA activity. The WAICP100^VL^ strain with the highest PrpD^VL^ expression showed the highest itaconate production (1.35 g/L) after 48 h of cultivation. Next, the TCA cycle and glyoxylate shunt were additionally activated by overexpression of citrate synthase and isocitrate lyase encoded by *gltA* and *aceA* for further flux amplification and to facilitate the anaplerotic reaction to maximize itaconate production, respectively^14,32^ (Fig. 4a, b). The expression of *aceA* was varied to determine the optimized flux distribution between the glyoxylate shunt and TCA cycle^14,32^. The itaconate titer was increased along with increased expression of *aceA* (Fig. 4a), and the WAICPG5 strain with the highest expression level of *aceA* showed a 1.52-fold increase in itaconate production (2.03 g/L) compared to the WAICP100^VL^ strain after 48 h of cultivation. Finally, itaconate production reached up to 3.13 g/L at 96 h; the yield was maintained at a significant level (0.31 g/g, 43.0% of theoretical maximum yield)^14^ throughout cultivation and was driven by the substantially increased acetate uptake rate (Fig. 4b)^14^. Also, it should be noted that other metabolites were not detected meaningfully by HPLC in the medium except itaconate and citrate as products. These results indicate that in addition to the strongly kinetically compartmentalized flux toward the itaconate, the flux distribution could be optimized for itaconate production.

**Fig. 5:**
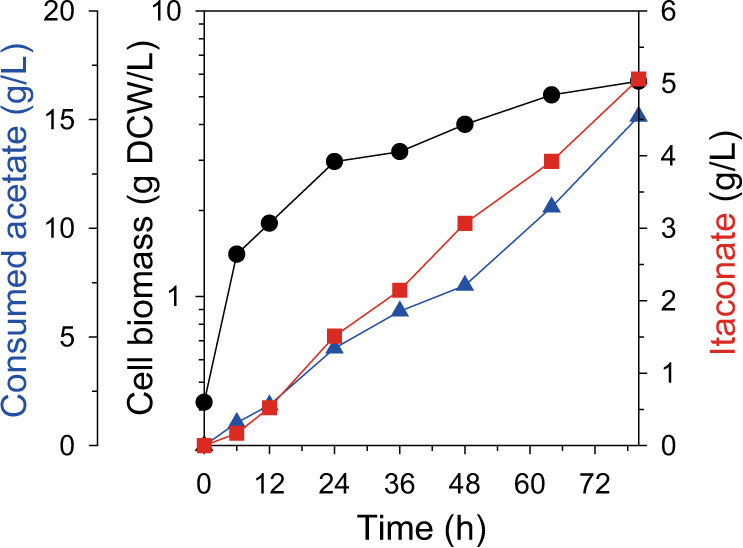
Fed-batch fermentation profile of WAICPG5. The left y-axis and y-offset represent the cell biomass (g DCW/L) and consumed acetate (g/L), respectively. The right y-axis indicates the production of itaconate (g/L). The x-axis denotes time (h). The representative fermentation profiles was plotted. Source data are provided as a Source Data file.

To further validate the potential of the WAICPG5 strain, 5 L-scale fed-batch fermentation was performed (Fig. 5). To obtain enough cell biomass at the initial stage (~8 h), the acetate concentration was kept low at 5 g/L and in the subsequent production stage, it was adjusted to 10 g/L. As a result, itaconate was rapidly produced until 80 h, up to 5.06 g/L. In addition, the yield could be maintained (0.33 g/g), similar to the flask-scale culture during the fermentation, which is equivalent to 46% of the theoretical maximum yield from acetate. Considering the characteristics of acetate as a carbon source^33,34^, these results are remarkably high, and it far exceeds the previous titer (3.57 g/L) and yield (0.09 g/g) of itaconate production from acetate^14^. We also noted that itaconate productivity decreased sharply after 80 h in our initial-stage reactor study, which is considered to be a spontaneous genetic escapee due to acetate toxicity^34,35^. This will be intensively addressed through future reactor study with various approaches, including preventing-escapee strategies^31,36^ and reactor-parameter study^37^.

### Applicability of kinetic compartmentalization to itaconate production

Although kinetic compartmentalization was successfully applied to the itaconate production from acetate, this strategy may not work for other carbon sources such as glucose as the cellular metabolism significantly differs between carbon sources^38,39^. Therefore, we validated the potential of our strategy to be broadly applied to the utilization of other carbon sources, and it was re-built for the most widely studied glucose-based production (Supplementary Data 1)^11,40^.

**Fig. 6:**
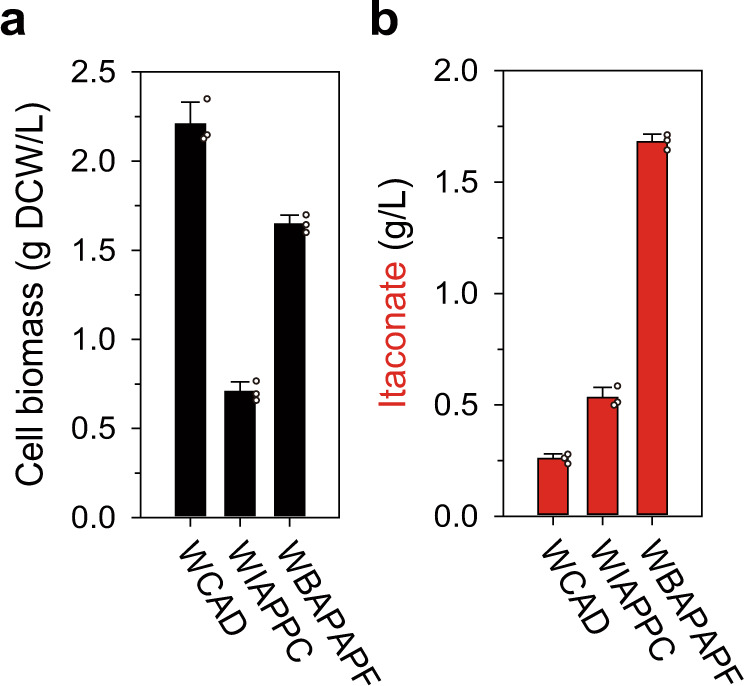
Application of kinetic compartmentalization approach for itaconate production from glucose. Comparison of the **a** cell biomass and **b** itaconate titer with glucose-utilizing strains after 60 h. The right y-axis indicates the cell biomass and production of itaconate (g/L), respectively. The x-axis denotes strains. Data were presented as mean values and error bars indicate the standard deviations from three biological replicates. White dots indicate actual data. Source data are provided as a Source Data file.

The WCAD strain was initially constructed, which harbors *cad* expressing-plasmid (pCAD) (Supplementary Data 1). Thereafter, the isocitrate dehydrogenase (encoded by *icd*) knockout strategy, which has been most widely and effectively utilized^11,40^, was preferentially applied. Then, phosphate acetyltransferase (*pta*), acetate kinase (*ackA*), and glucose-specific PTS component (*pts*) were additionally inactivated. Finally, phosphoenolpyruvate carboxylase (*ppc*) was overexpressed for the efficient anaplerosis^41^, resulting in WIAPPC strain (Supplementary Data 1) as a control strain. The WCAD strain (Supplementary Data 1) consumed most of the glucose (10 g/L) for cell biomass and the formation of acetate and then utilized secondary metabolites to produce itaconate (0.26 g/L, Fig. 6, Supplementary Fig. 11a). On the other hand, WIAPPC strain could consistently produce itaconate from the beginning and showed a 2.03-fold increase (0.53 g/L) compared to the WCAD strain, however, severe retardations in cell growth and glucose consumption were observed (Supplementary Fig. 11b) without additional TCA supplements (the culture medium already contains 2 g/L of yeast extract) due to TCA imbalance caused by *icd* knockout^42^.

In order to apply the kinetic compartmentalization strategy considering higher TCA flux exists from glucose compared to from acetate, the activities of competing enzymes of PrpD^VL^ were initially adjusted^43^; aconitate hydratase 1 (*acnA*) was inactivated, and the expression of aconitate hydratase 2 (*acnB*) was downregulated (Supplementary Data 1). As a result, the WBAPAPF strain in which PrpD^VL^ was introduced could efficiently produce itaconate (1.68 g/L, Fig. 6 and Supplementary Fig. 11c). These results correspond to a 6.46-fold increase compared to the WCAD strain and a 3.16-fold increase compared to the WIAPPC strain with a comparative *icd* knockout strategy. Besides increasing itaconate production, the WBAPAPF strain showed robust cell growth compared to the WIAPPC strain, suggesting that additional supplements such as yeast extract or glutamate are not required, unlike the previous strategy. Overall, our early-stage validation for glucose-based production further demonstrates the potential of our strategy. That is expected to be widely applicable to itaconate production systems in the future^44,45^.

## Discussion

In nature, efficiently designed spatial compartmentalization systems have been introduced to preserve substrate availability^13^ or prevent damage caused by toxic intermediates^15,46^. Naturally assembled proteinaceous organelles, including carboxysomes^47^ and metabolosomes^48^ have been detected even in some prokaryotes. However, in most prokaryotes, simultaneous reactions involving numerous substrates within a single space are determined only by the kinetic properties of the enzymes, which have evolved to be well-coordinated to maximize cell growth with high precision. Inspired by these characteristics, we developed a non-natural enzymatic reaction, resulting in successful kinetic compartmentalization in prokaryotes for itaconate production. Given that around 40–50% of enzymes with known functions have multiple substrates and 10–20% of these multi-substrate-specific enzymes can mediate consecutive reactions (Supplementary Fig. 12 and see Supplementary Data 2), our approach can be applied to other consecutive reactions to enable metabolic engineering of pathways for hardly obtainable intermediates.

Numerous studies of the heterologous production of itaconate have consistently focused on substrate availability^11,14,49^ but could not imitate or recapitulate the spatial compartmentalization strategy of its native producer, *A. terreus*, pumping out *cis*-aconitate into an independent space^13^. Therefore, we introduced kinetic compartmentalization to provide an efficient supply of *cis*-aconitate. Rather than using existing aconitase, which is highly reactive to *cis*-aconitate, the promiscuous enzyme PrpD was semi-rationally engineered and used to improve the itaconate production. We carried out the itaconate production in *E. coli* both using acetate, which is a non-preferred carbon source requiring intensive engineering for its efficient conversion^14^, and glucose, widely utilized in fermentation.

In addition to the modified catalytic characteristic of PrpD, the non-natural *cis*-aconitate synthesis reaction can be regulated by the expression level of the PrpD mutant. Overall, the itaconate titer was increased when a stronger promoter was used to express PrpD^VL^ (Supplementary Fig. 10), indicating that more carbon flux was kinetically compartmentalized according to PrpD^VL^ expression. Surprisingly, the resulting strain WAICPG5 produced up to 5.06 g/L itaconate at 46.0% of the theoretical maximum yield from acetate (Fig. 5), which showed a 2.86-fold increase in yield compared to the previous study^14^, indicating the efficient kinetic compartmentalization.

An itaconate-responsive screening system was successfully constructed in this study. The initial library was constructed to sufficiently cover all combinations; however, while determining the effect of each residue, double mutants with more desired characteristics were identified. In addition, the enriched population clearly contained mutants that improved itaconate production; however, not all mutants showed the desired phenotype. After the PrpD^VTL^ mutant dominates during the initial enrichment, hitchhikers may have been enriched together through crosstalk effects^50^. Nonetheless, we screened for effective mutants in four rounds of enrichment and observed an unintended decrease in catalytic efficiency toward *cis*-aconitate, supporting the effectiveness of the high-throughput screening system^22,25,26,51,52^. This method can be further applied in diverse strategies for itaconate production, including adaptive laboratory evolution, the evolution of *cis*-aconitate decarboxylase, and even the de novo itaconate production pathway.

## Methods

### Oligonucleotides and reagents

Oligonucleotides were obtained from Cosmo Genetech (Seoul, South Korea). For routine DNA manipulation, Takara PrimeSTAR™ HS DNA Polymerase (R040A, Shiga, Japan) was used for DNA amplification. Plasmid DNA and genomic DNA were extracted using the AccuPrep^R^ Nano-Plus Plasmid Mini Extraction Kit (K-3111G, Bioneer, Daejeon, South Korea) and GeneAll^R^ Exgene^TM^ Cell SV Kit (106-101, GeneAll, Seoul, South Korea), respectively. Enzymes, including ligase and restriction enzymes, and Gibson assembly were purchased from New England Biolabs (Ipswich, MA, USA). Other chemical reagents were obtained from Sigma-Aldrich (St. Louis, MO, USA) unless otherwise stated.

### Bacterial strains and plasmids

All bacterial strains and plasmids used in this study are listed in Supplementary Data 1. *Escherichia coli* Mach1-T1^R^ (Thermo Fisher Scientific, Waltham, MA, USA) was utilized as a cloning host, and the acid-tolerant *E. coli* W strain was utilized as an expression host^14^ and source of wild-type *prpD*. The WA, WAI, WAIPPC, and WBAPAPF strains were constructed using the Lambda-Red recombination system with plasmids pM_FKF, pKD46, and pCP20^53^. The pCDF_CAD and pET-P7 plasmid were used to construct pCAD and pCPHLF, respectively. And variants of the pCOGA plasmid (pCOGA, pCOGA1,3,4,5) were used to amplify the glyoxylate shunt from our previous study^14^. The itaconate-responsive screening system, a codon-optimized *itcR* fragment from *Y. pseudotuberculosis*, was synthesized and assembled with amplified pETduet-1 and *tetA* fragments^24,25^. pPRPD was constructed by assembling amplified pACYCduet-1 and *prpD* from *E. coli* W. Genetic variants of PrpD were obtained by site-directed mutagenesis and the Gibson assembly method^54^. The terminators and promoters for all vectors were obtained from the Registry of Standard Biological Parts (http://parts.igem.org). Synthetic 5′ untranslated regions were computationally designed using UTR Designer (http://sbi.postech.ac.kr/utr_designer)^55^.

### Cell cultivation and enrichment for screening

Cells were cultivated in modified minimal acetate medium containing 0.5 g/L MgSO_4_·7H_2_O (M2773), 2.0 g/L NH_4_Cl (A9434), 1.0 g/L NaCl (S5886), 2.0 g/L yeast extract (70161), and 100 mM potassium phosphate buffer (P8584 and P8709, pH 7.0)^14^. As a carbon source, 10 g/L neutralized acetate (A6283, pH 7.0) was added to the medium. To maintain the plasmids, antibiotics were added to the medium (50 µg/mL streptomycin, 50 µg/mL kanamycin, 34 µg/mL chloramphenicol, and 100 µg/mL ampicillin).

For itaconate production, single colonies of each strain were inoculated into 3 mL of medium in a 15 mL test tube. After 12 h, the cell cultures were inoculated into 3 or 20 mL of fresh medium in the test tube or 300-mL Erlenmeyer flasks, respectively, to an optical density at 600 nm (OD_600_) of 0.05. Isopropyl-β-d-thiogalactopyranoside (I5502) was initially added to a final concentration of 0.1 mM for induction. Cultures were performed in biological triplicate with continuous shaking (200 rpm) at 30 °C^14^. The pH was adjusted to 7.0 by adding an appropriate amount of 5 M HCl solution (H1758). Culture samples were periodically collected and stored at −80 °C for analysis. The theoretical maximum yield from acetate to itaconate was calculated to be 0.72 g/g referring to the stoichiometric balance of the previous study^14^. A 5 L-scale reactor study (Marado-PDA, BIOCNS, Daejeon, South Korea) was utilized. The recombinant strain was inoculated to 1.2 L of modified minimal medium containing 5 g/L of acetate. Sterile air was pumped at a flow rate of 5vvm and 0.1% (v/v) of antifoam 204 (A8311) was treated to prevent the foaming. Acetate concentration was intermittently added to be maintained above 10 g/L.

To validate the screening system and enrichment for screening PrpD mutants, we exerted selection pressure using tetracycline (T7660). The concentration of tetracycline varied from 0–50 mg/L to determine the initial selection pressure. The toxicity of itaconate was determined using various concentrations of itaconate (I29204) from 0–2 g/L. The mutant library was initially enriched with 7 mg/L of tetracycline and increased to 15 mg/L over four rounds of enrichment. All cultures except for the enrichment culture were conducted in triplicate.

### Analytical methods to detect cellular metabolites

Cell biomass (OD_600_) was measured using a UV-1700 spectrophotometer (Shimadzu, Kyoto, Japan), and the dry cell weight (DCW) was calculated by converting 1 unit of OD_600_ to 0.31 g/L^56^. Metabolites were measured using an Ultimate 3000 high-performance liquid chromatography system (Dionex, Sunnyvale, CA, USA). Filtered samples were analyzed using an Aminex HPX-87H column (Bio-Rad Laboratories, Hercules, CA, USA). In the mobile phase, 5 mM H_2_SO_4_ (SX1248) was used at a flow rate of 0.6 mL/min; the temperature of the column oven was maintained at 14 °C^29^. The refractive index and absorbance at a UV wavelength of 210 nm were monitored using a Shodex RI-101 detector (Shodex, Klokkerfaldet, Denmark) and a variable wavelength detector (Dionex).

### Characterization of enzyme kinetics

To validate the enzyme kinetics, cells were cultivated for 9 h after induction, and cell pellets were resuspended in 40 mM Tris-HCl buffer (Bioneer, C-9006, pH 8.0). The cells were lysed using a Qsonica sonicator (Sonics & Materials, Newtown, CT, USA) for 3 min. To avoid oxidation of an iron-sulfur cluster of enzymes, dithiothreitol (43816) and (NH_4_)_2_Fe(SO_4_)_2_ (09719) were added to final concentrations of 2.5 and 0.25 mM, respectively^8^. The cell lysates were centrifuged for 10 min at 13,000×*g* at 4 °C. The supernatants were utilized to purify the 6X His-tagged enzymes with a MagListo^TM^ His-tagged protein purification kit (Bioneer, K-7200) under anaerobic conditions to prevent the inactivation of aconitase activity^8^. The elutes were treated with 1 mM dithiothreitol, 0.14 mM (NH_4_)_2_Fe(SO_4_)_2_, and 0.12 mM Na_2_S (407410) to prevent the oxidization of iron-sulfur clusters. The amount of purified enzyme was quantified and adjusted to the same amount using the Bradford assay (B6916).

The enzyme assay was conducted in 2 mM Tris-HCl buffer with varying amounts of substrate: 1, 2, 5, 10, and 20 mM for citrate (251275); 0.05, 0.1, 0.2, 0.5, and 1.0 mM for *cis*-aconitate (A3412); 0.1, 0.2, 0.4, 0.6, and 1 mM for 2-methylcitrate (59464). This reaction was conducted at 37 °C for 10 min followed by the inactivation of the enzyme at 90 °C for 5 min. The samples were analyzed using a high-performance liquid chromatography system with an XTerra^R^ RP18 column and the Aminex HPX-87H column (Bio-Rad Laboratories, Hercules, CA, USA). H_2_SO_4_ (5 mM) was utilized as the mobile phase at a flow rate of 0.6 mL/min. Absorbance was monitored at a UV wavelength of 215 nm. All assays were conducted in triplicate.

### Structural analysis for PrpD mutant library design

To identify the catalytic sites of PrpD, a structural model of PrpD was first generated using the structure of the homologous protein MmgE (PDB code: 5MUX)^27^ from *B. subtilis*. From the ligand of 5MUX, the catalytic site of MmgE was identified, and well-conserved residues were found in both PrpD and MmgE (Supplementary Fig. 3)^57^. A 3D model of 2-methylcitrate was generated from the downloaded SDF format of (2 *S*,3 *S*)−2-methylcitrate from PubChem. The SDF file was converted to PDB format using the online SMILES translator and structure file generator (https://cactus.nci.nih.gov/translate/). Finally, the 3D model of 2-methylcitrate was aligned with the ligand of 5MUX using LS-Align. From this final model structure, the distance between the catalytic sites of PrpD and nearby residue from the methyl group of 2-methylcitrate was calculated (Supplementary Table 1).

Among the residues in the catalytic site, those key for catalytic reactions and important for the interaction with the carboxyl group of citrates were preserved to maintain catalytic activity. For example, histidine often acts as a proton donor and acceptor, which is critical in the catalytic reaction, and arginine is an open-form salt bridge with the anion form of a carboxyl group. Hydrophobic and small residues near the methyl group of 2-methylcitrate were selected as target residues for the mutant library.

### Docking simulations of the mutants

Both the PrpD natural substrate 2-methylcitrate (PubChem ID: 5460420) and citrate (PubChem ID: 31348) were docked into the active pocket of the PrpD enzyme using the crystallographic structure of apo-protein 2-methylcitrate dehydratase from *E. coli* (PDB: 1SZQ [https://www.rcsb.org/structure/1SZQ])^27^ using Chimera software^58^. This protein structure included two chains, one of which was deleted before the docking simulation. The protein was prepared by eliminating water molecules and adding hydrogen and charge. The ligands were also charged before analysis. As the presence of the methyl group of 2-methylcitrate is an important factor in the substrate specificity of PrpD, the orientation of this group was evaluated in all docking simulations along with the number of hydrogen bonds between the ligand and protein residues resulting from each analysis.

### Characterization of multi-substrate and consecutive enzymes

We collected the enzyme reactions of *E. coli* from KEGG (https://pubmed.ncbi.nlm.nih.gov/10592173/) and BioCyc (https://pubmed.ncbi.nlm.nih.gov/29447345/). To characterize enzymes that have multiple substrate specificities, we counted the number of known reactions for each enzyme. If the enzyme is involved in more than one enzymatic reaction, we categorized them as multiple substrate-specific enzymes. Then, we further characterize enzymes that can conduct consecutive reactions by themselves by checking if the same enzyme can take their product in one reaction as substrate in the other reactions. During the analysis we excluded the following molecules because they can work as cofactors or common substrates: “H2O”, “H+”, “ATP”, “GTP”, “CTP”, “TTP”, “ADP”, “GDP”, “CDP”, “TDP”, “NADH”, “NADH+”, “NADPH”, “CO2”, “NAD”, “NADP+”, “AMP”, “GMP”, “CMP”, “TMP”, “Fe2+”, “Fe3+”, and “phosphate”.

In order to find an enzyme that can be used to make kinetic compartmentalization, same as our strategy, we first choose consecutive enzyme reactions of *E. coli* from KEGG. Then we searched the assigned EC (Enzyme Commission) number of enzymes that facilitate such consecutive reactions. If the enzymes have more than two EC numbers, we took the first EC number and manually find the other enzyme from the super-class of the selected EC number with a similar substrate structure. As EC number is defined as enzyme classes, subclasses have similar functions. For example, AcnB is assigned both EC:4.2.1.3 and EC:4.2.1.99, so we searched the enzymes that have similar substrate structures to AcnB. PrpD (EC: 4.2.1.79) was selected as it converts 2-methylcitrate that has a similar structure to citrate, a substrate of AcnB. The suggested enzyme list is provided as Supplementary Data 2.

### Reporting summary

Further information on research design is available in the Nature Research Reporting Summary linked to this article.

## Supplementary information

Supplementary Information

Description of Additional Supplementary Files

Supplementary Data 1

Supplementary Data 2

Reporting Summary

## Data Availability

Data supporting the findings of this work are available within the paper and its Supplementary Information files. A reporting summary for this Article is available as a Supplementary Information file. Source data are provided with this paper.

## Source data

Source Data
