# Supplementary material for: Kinetic compartmentalization by unnatural reaction for itaconate production
Source: Nat Commun. 2022 Sep 12;13:5353. doi: 10.1038/s41467-022-33033-1 (PMC9468356; doi:10.1038/s41467-022-33033-1)
Supplement: Supplementary file 1 — Supplementary Information [file 41467_2022_33033_MOESM1_ESM.pdf]

Kinetic compartmentalization by unnatural reaction for itaconate production

Ye *et al.*

Supplementary Table 1. Distance from the methyl group of second carbon of 2-methylcitrate.

Amino acid	Residue number (MmgE)	Residue number (PrpD)	Atom name	Distance (Å)
HIS	104	112	NE2	3.38
TRP	102	110	CB	3.46
HIS	163	171	CE1	3.90
GLY	103	111	N	4.06
HIS	163	171	ND1	4.11
HIS	104	112	CD2	4.14
GLU	282	290	OE2	4.16
HIS	104	112	CE1	4.23
TRP	102	110	C	4.24
GLY	103	111	CA	4.38
TRP	102	110	CG	4.45
TRP	102	110	CA	4.49
TRP	102	110	O	4.75
TRP	102	110	CE3	4.94
HIS	163	171	NE2	4.95
TRP	102	110	CD2	4.95

Residue numbering was based on 2-methylcitrate dehydratase (MmgE) from Bacillus subtilis (PDB code: 5MUX)1. Atoms with atomic distances less than 5 Å between the predicted PrpD residue and methyl group of second carbon of 2-methylcitrate are shown.

Supplementary Table 2. List of isolated mutants from the enriched population.

Name	110th	111th	331st	Frequency
pPRPD	Trp	Gly	Ile	Wild-type
$pPRPD^{CHP}$	Cys	His	Pro	1
$pPRPD^{SLQ}$	Ser	Leu	Gln	1
$pPRPD^{SSN}$	Ser	Ser	Asn	1
$pPRPD^{VLR}$	Val	Leu	Arg	4
$pPRPD^{VTL}$	Val	Thr	Leu	3
Total				10

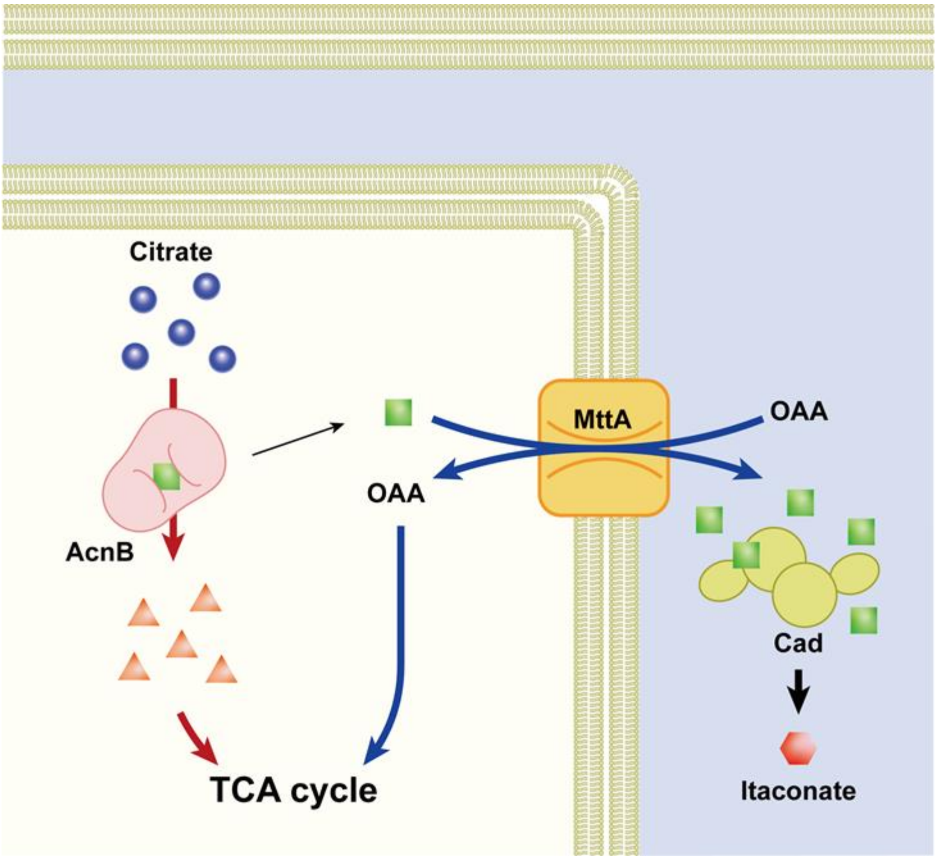

Supplementary Figure 1. Spatial compartmentalization in *Aspergillus terreus* for itaconate production. Citrate (blue); isocitrate (orange); *cis*-aconitate (green); itaconate (red).

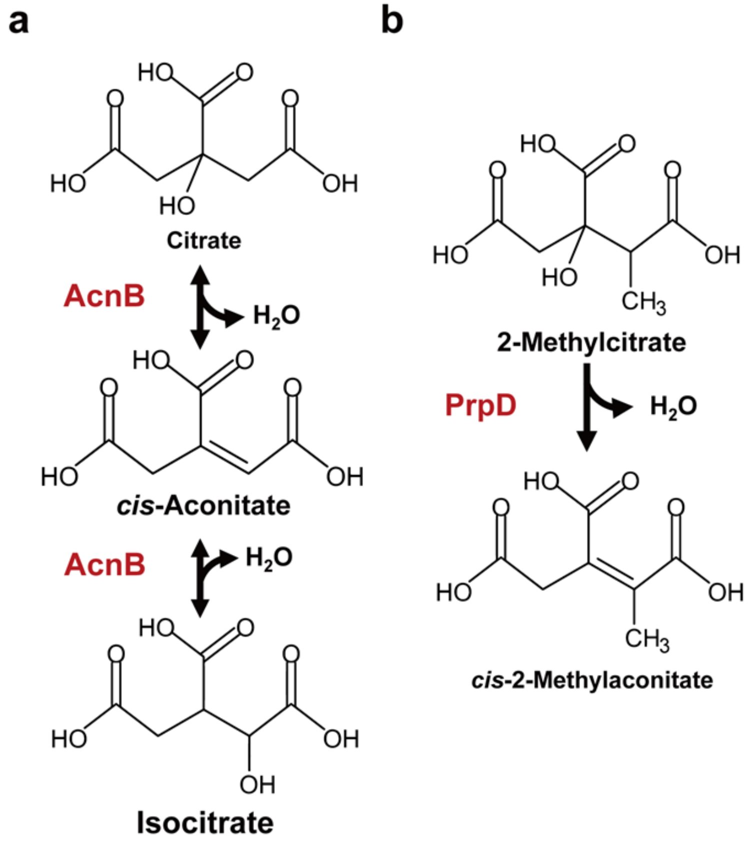

Supplementary Figure 2. Enzymatic reaction of (a) AcnB and (b) PrpD.

```
CLUSTAL O(1.2.4) multiple sequence alignment

5MUX            --------MPKTDRVIEEITDYVLEKEITSAEAYTTAGHVLLDTLGCGILALRYPECTKL 52
PRPD_ECOLI      MSAQINNIRPEFDREIVDIVDYVMNYEISSKVAYDTAHYCLLDTLGCGLEALEYPACKKL 60
                         *: ** * :*.***:: **:*  ** ** : ********: **.** *.**

5MUX            LGPIVPGTTVPNGSKVPGTSYVLDPVRAAFNIGCMIRWLDYNDTWLAAEWGHPSDNLGGI 112
PRPD_ECOLI      LGPIVPGTVVPNGVRVPGTQFQLDPVQAAFNIGAMIRWLDFNDTWLAAEWGHPSDNLGGI 120
                ********.**** :****.: ****:******.******:*******************

5MUX            LAAADYVSRVRLSEGKEPLTVRDVLEMMIKAHEIQGVLALENSLNRVGLDHVLFVKVATT 172
PRPD_ECOLI      LATADWLSRNAVASGKAPLTMKQVLTAMIKAHEIQGCIALENSFNRVGLDHVLLVKVAST 180
                **:**::**  ::.** ***:::**  ********* :*****:*********:****:*

5MUX            AVAAKLLGGGREEIKNALSNAWIDNAALRTYRHSPNTGSRKSWPAGDATSRGVHLALMSL 232
PRPD_ECOLI      AVVAEMLGLTREEILNAVSLAWVDGQSLRTYRHAPNTGTRKSWAAGDATSRAVRLALMAK 240
                **.*::**  **** **:* **:*. :******:****:**** *******.*:****:

5MUX            KGEMGYPTALSAPGWGFQDVLFNKKEIKLARPLDAYVMENVLFKVSYPAEFHAQTAAESA 292
PRPD_ECOLI      TGEMGYPSALTAPVWGFYDVSFKGESFRFQRPYGSYVMENVLFKISFPAEFHSQTAVEAA 300
                .******:**:** *** ** *: :.::: ** .:*********:*:*****:***.*:*

5MUX            VILHPQVK---NRIDEIDRVVIRTHESAIRIIDKKGPLHNPADRDHCLQYITAIGLLFGD 349
PRPD_ECOLI      MTLYEQMQAAGKTAADIEKVTIRTHEACIRIIDKKGPLNNPADRDHCIQYMVAIPLLFGR 360
                : *: *::   :   :*::*.*****:.********:*******:**:.** ****

5MUX            ITAQHYEAETANDPRIDKLRDKMEVTENKTYTEDYLKPDKRSISNAVQVHFKDGTSTEMV 409
PRPD_ECOLI      LTAADYEDNVAQDKRIDALREKINCFEDPAFTADYHDPEKRAIANAITLEFTDGTRFEEV 420
                :** .** :.*:* *** **:*::  *: ::* ** .*:**:*:**: :.*.***  * *

5MUX            ECEFPLGHRFRREEAVPKLLEKFSDNLKTHFPDKQHKHIYERCTSYETLQTMRVNEFVDM 469
PRPD_ECOLI      VVEYPIGHARRRQDGIPKLVDKFKINLARQFPTRQQQRILEVSLDRARLEQMPVNEYLDL 480
                  *:*:**  **::.:***::**. **  :** :*:::* * . .   *: * ***::*:

5MUX            FCM      472
PRPD_ECOLI      YVI      483
```

Supplementary Figure 3. Sequence alignment[2] between 2-methylcitrate dehydratase (MmgE) from *Bacillus subtilis* and PrpD from *Escherichia coli*. Sequences were obtained from the crystal structure (PDB code: 5MUX)[1] and Swiss-Prot (P77243|PRPD_ECOLI). Red and bold characters indicate the catalytic sites defined from 5MUX crystal structure based on 4 Å distance from L-Tartaric acid ligand. All catalytic sites were conserved in both MmgE and PrpD.

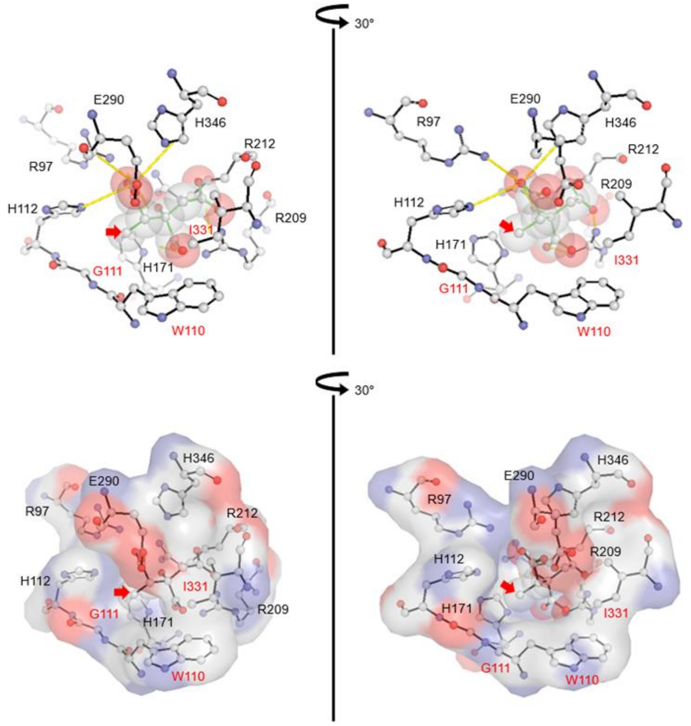

Supplementary Figure 4. Specifying the mutagenesis residues interacting with methyl group of 2-methylcitrate by docking simulation between 2-methylcitrate and PrpD from *E. coli*. In the upper panel of figure, the atomic connections between C-C and C-O of 2-methylcitrate were shown as green lines and the polar contacts were depicted as yellow lines. The red arrow indicates the methyl group of 2-methylcitrate and it is located toward to W110 and G111. H171 is located in the bottom of the substrate. The lower panel of figure shows the surface representation of pocket residues. G111, W110, and I331 residues make hydrophobic pocket. The residue numbering is based on PrpD from *E. coli*.

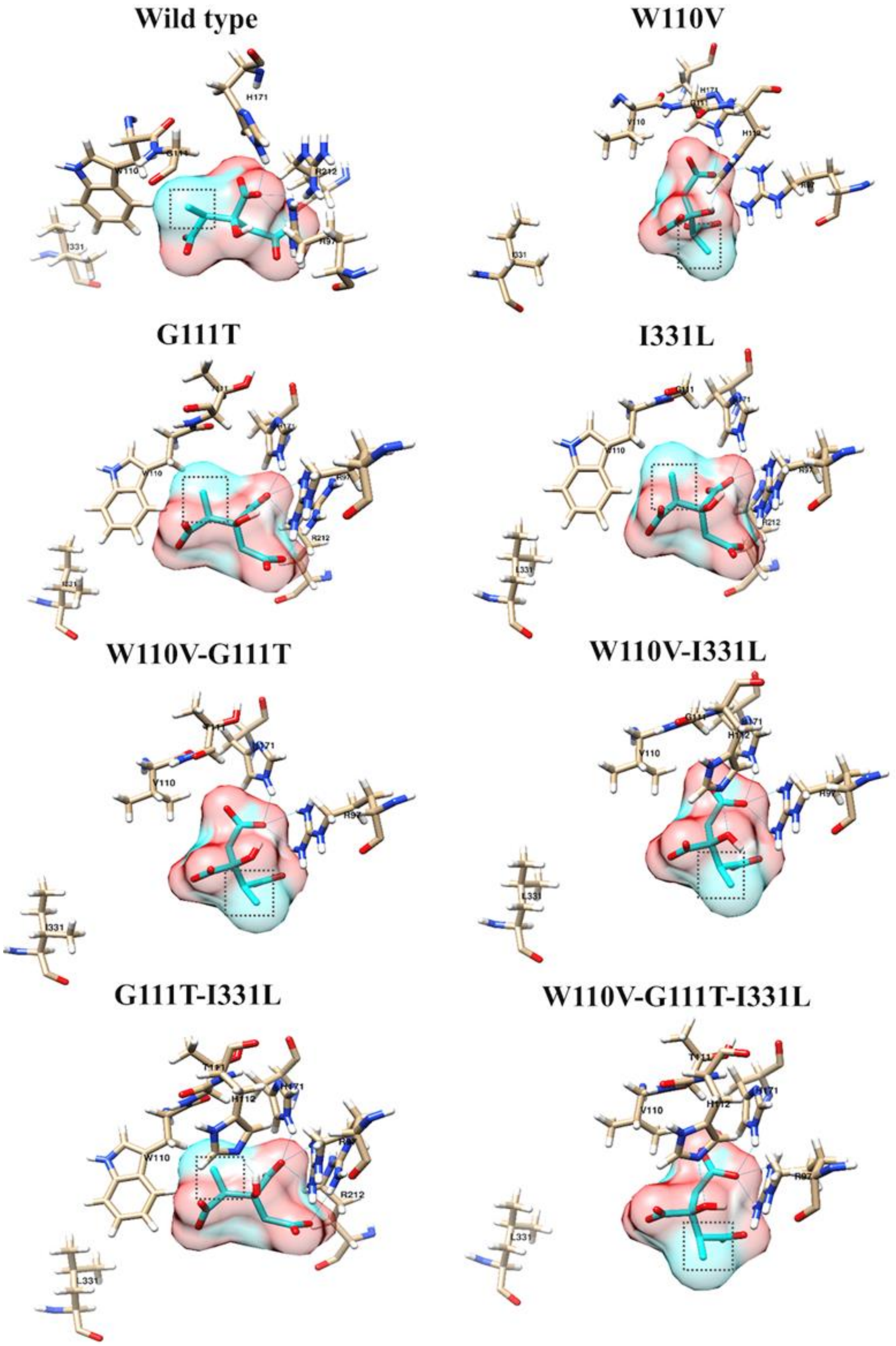

Wild type
W110V
G111T
I331L
W110V-G111T
W110V-I331L
G111T-I331L
W110V-G111T-I331L

Supplementary Figure 5. Docking simulation of 2-methylcitrate interactions into the active pocket of PrpD enzyme. The predicted interactions of 2-methylcitrate into the active site of PrpD (PDB of the apo-form of the protein: 1SQZ) are shown for the wild-type and different combinations of mutations (single, double, and triple), involving three residues predicted to be involved in substrate specificity. Carbon atoms of active site residues involved in the interactions are colored light grey, compared to carbon atoms of the ligand which are colored cyan. Oxygen atoms are colored as red, nitrogen as blue, and polar hydrogens as white. Nonpolar hydrogens are hidden. Solid blue lines represent hydrogen bonds. The dashed black box highlights the methyl group of 2-methylcitrate.

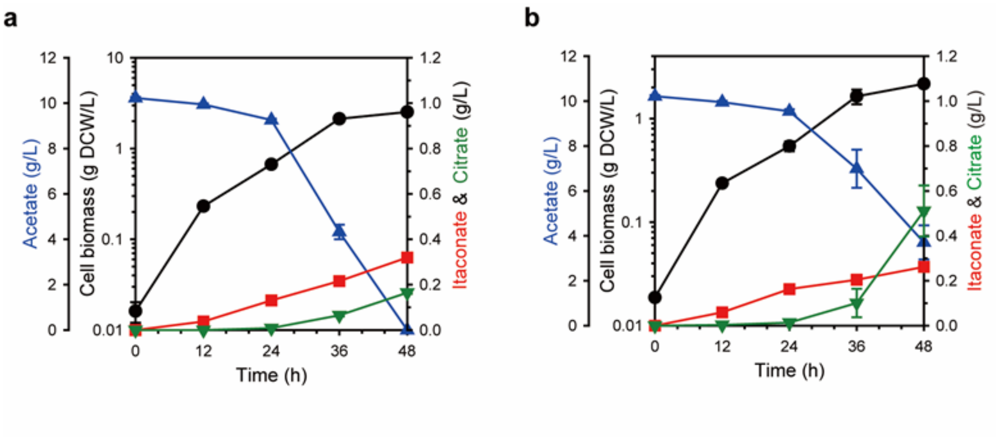

Supplementary Figure 6. Fermentation profiles of (a) WCI and (b) WAIC strains. The left y-axis and y-offset represent the cell biomass (g DCW/L) and acetate (g/L), respectively. The right y-axis indicates the production of itaconate and citrate (g/L). The x-axis denotes time (h). Circles, cell biomass; up-triangles, acetate; squares, itaconate; down-triangles, citrate. Data are presented as mean values and error bars indicate the standard deviations from three biological replicates. Source data are provided as a Source Data file.

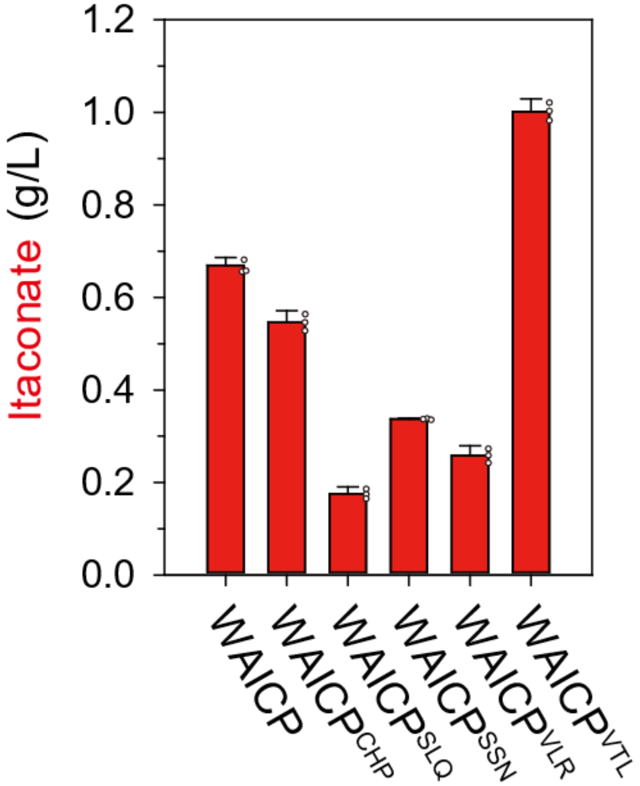

Supplementary Figure 7. Comparison of itaconate production with enriched PrpD mutants. Cultures were conducted on a test tube scale for 48 h. Data are presented as mean values and error bars indicate the standard deviations from three biological replicates. White dots indicate actual data. Source data are provided as a Source Data file.

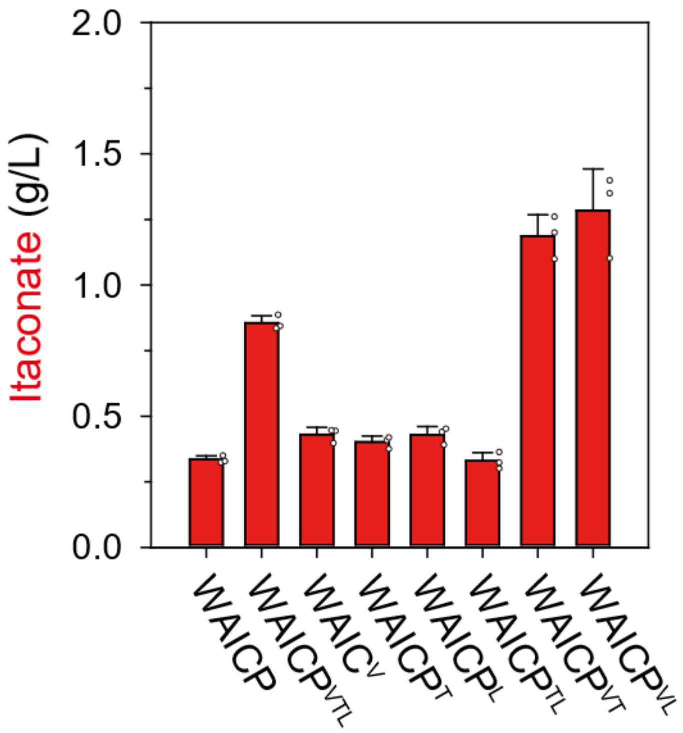

Supplementary Figure 8. Comparison of itaconate production with wild-type, PrpD^{VTL} and each combination of single- and double-mutant. Cultures were conducted on a flask scale for 48 h. Data are presented as mean values and error bars indicate the standard deviations from three biological replicates. White dots indicate actual data. Source data are provided as a Source Data file.

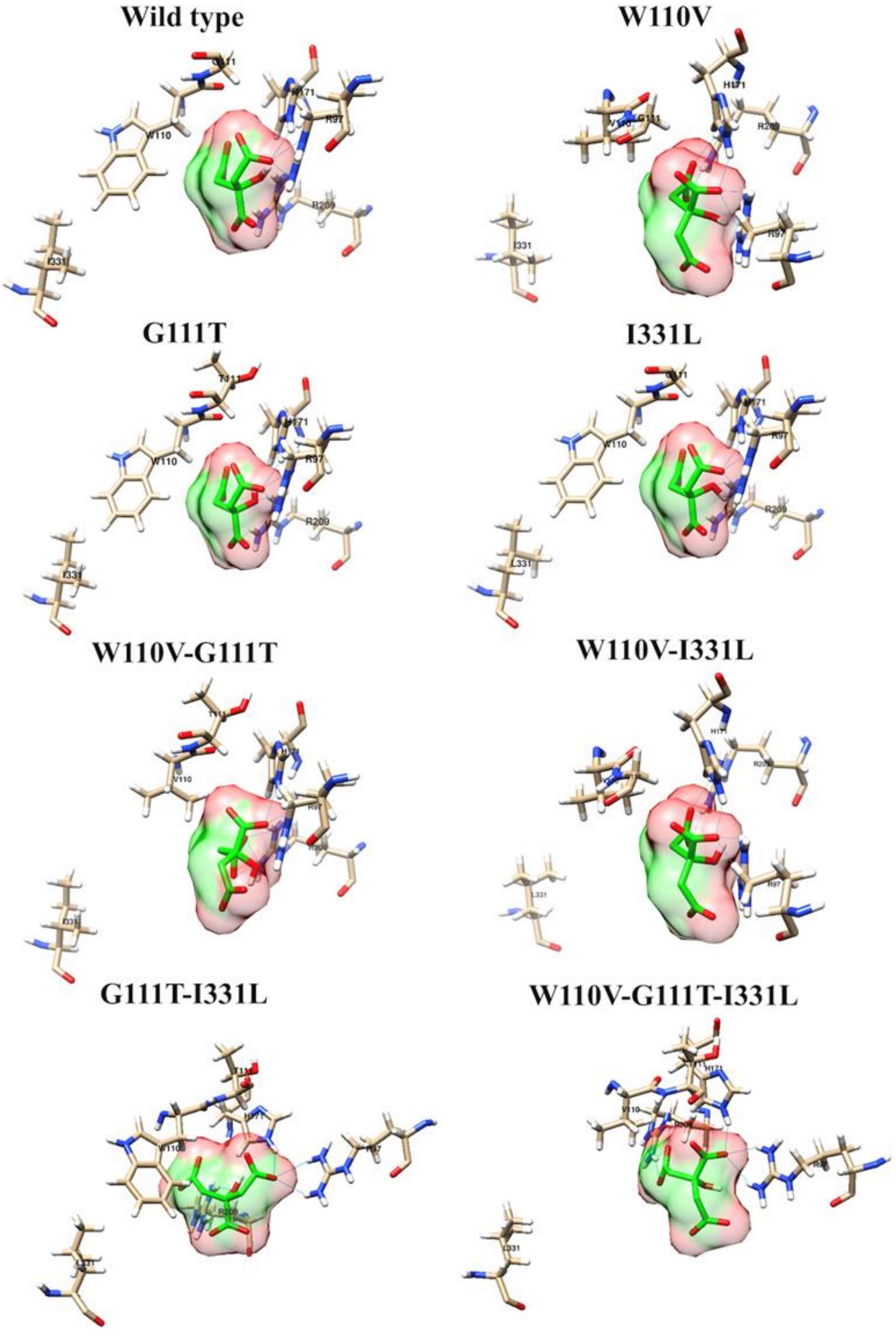
Wild type
W110V
G111T
I331L
W110V-G111T
W110V-I331L
G111T-I331L
W110V-G111T-I331L

Supplementary Figure 9. Docking simulation of citrate interactions into the active pocket of PrpD enzyme. The predicted interactions of citrate into the active site of PrpD (PDB of the apo-form of the protein: 1SQZ) are shown for the wild-type and different combinations of mutations (single, double, and triple), involving three residues predicted to be involved in substrate specificity. Carbon atoms of the active site residues involved in the interactions are colored as light grey, compared to carbon atoms of the ligand which are colored as light green. Oxygen atoms are colored as red, nitrogen as blue, and polar hydrogens as white. Nonpolar hydrogens are hidden. Solid blue lines represent hydrogen bonds.

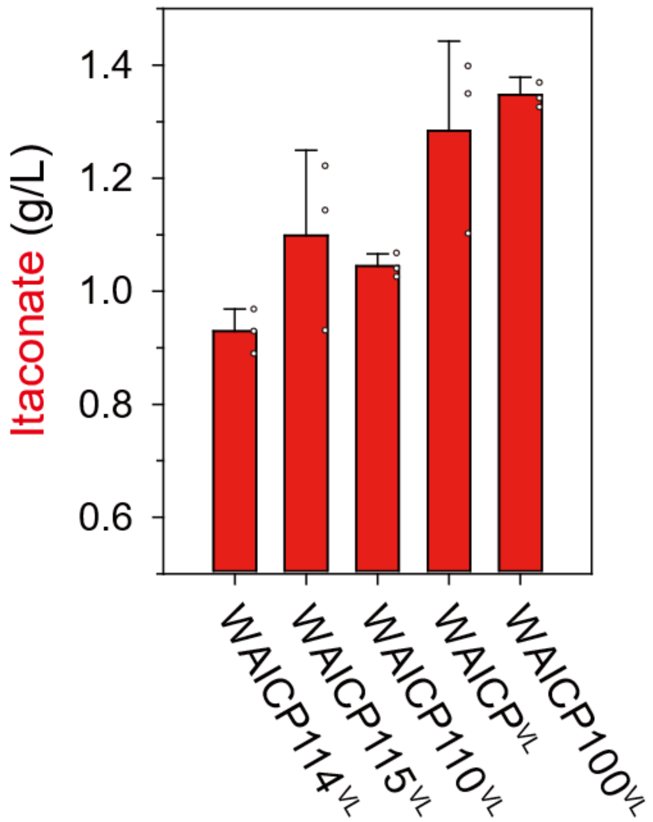

Supplementary Figure 10. Comparison of itaconate production of PrpD^{VL} under different strength of synthetic promoters. Cultures were conducted on a flask scale for 48 h. Data are presented as mean values and error bars indicate the standard deviations from three biological replicates. White dots indicate actual data. Source data are provided as a Source Data file.

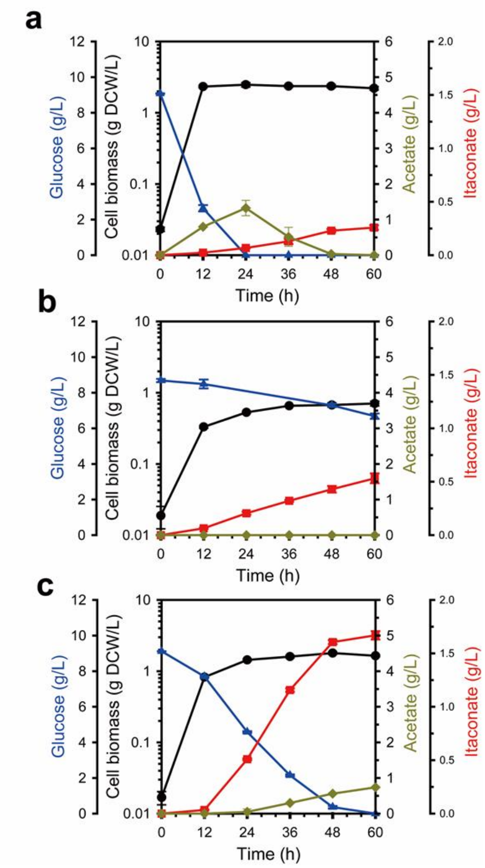

Supplementary Figure 11. Fermentation profiles of (a) WCAD, (b) WIAPPC and (C) WBAPAPF to validate the effect of kinetic compartmentalization. The left y-axis and y-offset represent the cell biomass (g DCW/L) and glucose (g/L), respectively. The right y-axis and y-offset indicates the production of acetate and itaconate (g/L), respectively. The x-axis denotes time (h). Circles, cell biomass; up-triangles, glucose; squares, itaconate; diamond, acetate. Data are presented as mean values and error bars indicate the standard deviations from three biological replicates. Source data are provided as a Source Data file.

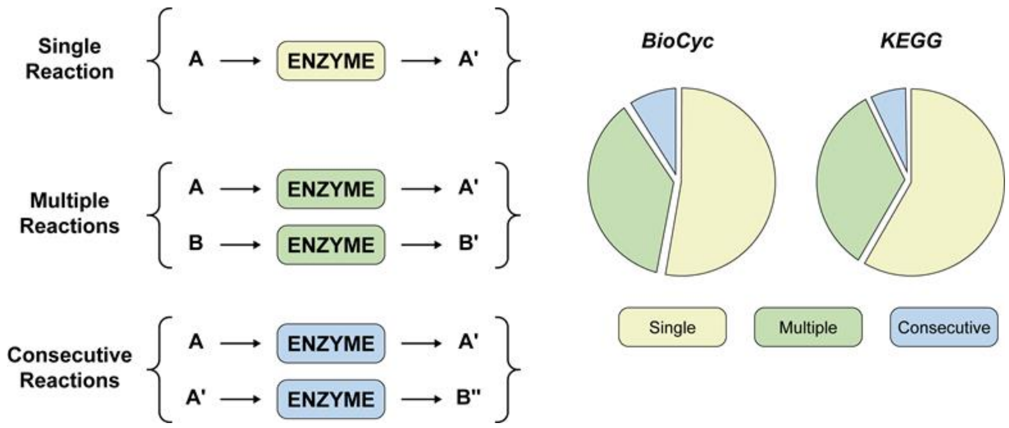

Supplementary Figure 12. The ratio of enzymes known to have multiple reactions or consecutive reactions registered in BioCyc and KEGG. Consecutive reactions were not counted as multiple reactions in this graph.

Supplementary references

1. H.M. Berman, J. Westbrook, Z. Feng, G. Gilliland, T.N. Bhat, H. Weissig, I.N. Shindyalov, P. E. B. The Protein Data Bank. *Nucleic Acids Res.* **28**, 235–242 (2000).

2. Sievers, F. *et al.* Fast, scalable generation of high-quality protein multiple sequence alignments using Clustal Omega. *Mol. Syst. Biol.* **7**, (2011).
