# Supplementary material for: Kinetic compartmentalization by unnatural reaction for itaconate production
Source: Nat Commun. 2022 Sep 12;13:5353. doi: 10.1038/s41467-022-33033-1 (PMC9468356; doi:10.1038/s41467-022-33033-1)
Supplement: Supplementary file 2 — Description of Additional Supplementary Files [file 41467_2022_33033_MOESM2_ESM.pdf]

Description of Additional Supplementary Files

File Name: Supplementary Data 1
Description: Bacterial strains and plasmids used in this study.

File Name: Supplementary Data 2
Description: Enzyme candidates that can used for kinetic compartmentalization
